# Biodegradability of Trimethylbenzene Isomers under Denitrifying and Sulfate-Reducing Conditions

**DOI:** 10.3390/ijerph16040615

**Published:** 2019-02-20

**Authors:** Thomas Fichtner, Axel Rene Fischer, Christina Dornack

**Affiliations:** Institute of Waste Management and Circular Economy, Technische Universität Dresden, 01796 Pirna, Germany; axel_rene.fischer@tu-dresden.de (A.R.F.); christina.dornack@tu-dresden.de (C.D.)

**Keywords:** trimethylbenzene, anaerobic biodegradation, nitrate, sulfate, microcosms, column experiments, BTEX

## Abstract

Trimethylbenzene (TMB) isomers (1,2,3-TMB, 1,2,4-TMB, and 1,3,5-TMB) are often used as conservative tracers in anaerobic, contaminated aquifers for assessing BTEX (benzene, toluene, ethylbenzene, xylenes) biodegradation at field sites. However, uncertainties exist about the behavior of these compounds under anaerobic conditions. For this reason, the influence of various parameters (temperature, residence time) on the biodegradability of TMB isomers was investigated under denitrifying and sulfate-reducing conditions in microcosms and 1D-column experiments. Soil and groundwater contaminated with a cocktail of aromatic hydrocarbons including the TMB isomers, both collected from an industrial site in Berlin, Germany, were used for the laboratory investigations. A continuous and complete biodegradation of 1,3,5-TMB and 1,2,4-TMB under denitrifying conditions was observed independent of realized temperature (10–20 °C) and residence time. Biodegradation of 1,2,3-TMB started after longer lag-phases and was not continuous over the whole experimental period; a strong dependence on temperature and residence time was identified. The biodegradability of all TMB isomers under sulfate-reducing conditions was continuous and complete at higher temperatures (20 °C), whereas no degradation was observed for lower temperatures (10 °C). First-order biodegradation rate constants ranged from 0.05 to 0.21 d^−1^ for 1,3,5-TMB and 1,2,4-TMB and from 0.01 to 0.11 d^−1^ for 1,2,3-TMB.

## 1. Introduction

Trimethylbenzene (TMB) isomers are common components of fuels as well as mixed hydrocarbon solvents. The individual representatives 1,2,3-TMB, 1,2,4-TMB, and 1,3,5-TMB often can be found together with other aromatic hydrocarbons such as benzene, toluene, ethylbenzene, and xylenes (BTEX). Soil and groundwater at many sites worldwide are contaminated with these compounds caused by leaking underground storage tanks or accidents in handling [1,2]. They are considered to be of major concern for aquatic organisms and human health, due to their high water solubility, mobility, and toxicity [1]. 

Anaerobic biodegradation processes usually play an important role in removing the three TMB isomers, due to the often limited oxygen availability in mineral oil-contaminated soils and aquifers [2,3,4]. An elimination of the substituents as well as an activation and destabilization of the aromatic ring by microorganisms are prerequisites for the occurrence of TMB-degradation. Moreover, the availability of terminal electron acceptors for the pollutant-degrading microorganisms is required for these processes to take place. TMB compounds are activated by the radical-based addition of a fumarate to the methyl groups and mineralized to carbon dioxide via the intermediate benzoyl-CoA [5,6].

The biodegradation of 1,2,4-TMB under denitrifying conditions was detected in the laboratory [2,3,7,8] as well as in the field [9,10] by several working groups. On the other hand, much less successful results have been obtained for the biodegradation of 1,3,5-TMB under similar laboratory conditions [2,3,7] and even less for 1,2,3-TMB [3]. The biodegradation of 1,2,4-TMB under sulfate-reducing conditions was detected by previous studies [11,12], whereas biodegradation of 1,2,4-TMB under iron/sulfate-reducing conditions was observed by Zheng et al. [13]. A degradation of 1,3,5-TMB was proven by previous studies [14,15]. Currently, there is no evidence of biodegradation for 1,2,3-TMB under sulfate-reducing conditions. The poor biodegradability of 1,2,3-TMB under anaerobic conditions can be explained by the structure of the TMB isomers. Destabilization of the aromatic ring and thus the biodegradation of the TMB isomers is different due to the grouping of the methyl groups. The destabilization of the aromatic ring is most difficult for 1,2,3-TMB because of the vicinal structure, the bonding of the three methyl groups to neighboring carbon atoms.

The three TMB isomers are often used as conservative tracers in anaerobic, contaminated aquifers [16,17] for assessing BTEX biodegradation at field sites. Their Henry’s Law constants and soil sorption coefficients are similar to BTEX, and they are known to be recalcitrant under anaerobic conditions, as reported by various previous investigations [17,18,19,20,21]. However, there are uncertainties and doubts about the use of the TMB isomers as conservative tracers in anaerobic, contaminated aquifers for assessing BTEX biodegradation. This is resulting from the small number of realized experiments and the contradictory results about the anaerobic biodegradation of TMB isomers under denitrifying and sulfate-reducing conditions. 

Therefore, the aim of this paper is it to identify the parameters and processes influencing the biodegradation of TMB isomers the most at a contaminated site. Laboratory experiments were performed to determine the relationship between different boundary conditions, such as redox conditions, temperature, and residence time, and the biodegradability of these substances by indigenous microorganisms. Further knowledge on this topic could be helpful when deciding about the enhancement of natural attenuation processes at sites contaminated with TMB. Based on the results, statements on the use of the TMB isomers as conservative tracers for assessing BTEX biodegradation at such sites can be made, which could be helpful in preventing the underestimation of BTEX biodegradation.

## 2. Materials and Methods 

### 2.1. Site Description

The investigated site is located in the north-western part of the German capital Berlin, in the Spandau district. The location was used as a storage place for petroleum products, primarily fuel derivatives, from 1920 to 1976. Additionally, a tar distillation facility was operated on this site. Contaminations of soil and groundwater with gasoline hydrocarbons are present at the site. The actual contamination of soil and groundwater with gasoline hydrocarbons has its origin in leaking underground storage tanks and surface spills, where mixtures of refined petroleum hydrocarbons in the form of fuel and tar with a variety of compounds and structural isomers were handled. Major components are the BTEX compounds (benzene, toluene, ethyl benzene, and xylene isomers), the three TMB isomers, polycyclic aromatic hydrocarbons (PAH), and alkyl phenols. Investigations at the site revealed that the main contamination is localized in the upper aquifer, which is separated from the deeper second aquifer by a glacial till. The downstream contamination plume is relatively long (250 m) but narrow (max. 80 m). The upper aquifer is characterized by quaternary sediments mainly composed of fine to medium sand. The depth to the water table is about 2 to 3 m and the thickness of the aquifer averages from 7 to 8 m. The groundwater flows with a low gradient (about 0.05 %) in a south-western direction.

### 2.2. Aquifer Material and Medium

Uncontaminated soil and contaminated groundwater collected from the investigated site (Table 1) were used for the microcosm and column experiments. The average concentrations of BTEX (12.1 mg/L) and TMB (1.3 mg/L) in the groundwater are in the same range as for other mineral oil-contaminated sites reported by previous studies [2,3].

Specific stainless steel containers were used to transport groundwater under strict anaerobic conditions from the site to the laboratory. The tanks were equipped with gas sampling bags to prevent evaporation of volatile compounds and were flushed with nitrogen to remove the oxygen before sampling. The macro-nutrient phosphate (Na_2_HPO_4_ origin) and trace elements (Table 2) were added to the used original contaminated groundwater to provide ideal conditions for the microorganisms. The components of the trace element solution according to Atlas [22] have been extended to the elements magnesium, tungsten, selenium, and aluminum.

### 2.3. Setup of the Microcosm Experiments

Cultures of indigenous microorganisms selected from the aquifer were enriched and used as inoculum. The incubation took place over a period of approximately 130 days at a constant temperature (20 °C). Sampling procedures were accomplished under strictly anaerobic conditions. The most active batches that degraded most of the site relevant pollutants were unified and provided the basis for the biodegradation experiments. Microcosms were prepared in 600-ml glass bottles in a glove box to ensure anaerobic conditions (nitrogen-helium atmosphere). Each of them contained 200 mL of contaminated groundwater from the site stored under strictly anaerobic conditions, 100 g of uncontaminated sediment from the site, and 35 mL of inoculum from the enrichment. Microcosms with the addition of the electron acceptors nitrate (KNO_3_ origin) as well as sulfate (Na_2_SO_4_ origin) were prepared for the determination of biodegradation under denitrifying as well as sulfate-reducing conditions. Furthermore, macro-nutrient phosphates as well as the trace elements were added to each microcosm (Table 3). The experiment included five active and three poisoned batches per redox condition, whereby the poisoned controls contained NaN_3_. The bottles were stored at 10 °C during the experimental period of 182 days (nitrate microcosms)/216 days (sulfate microcosms) to reproduce aquifer conditions. 

### 2.4. Setup of the Column Experiment 

**Figure 1:**
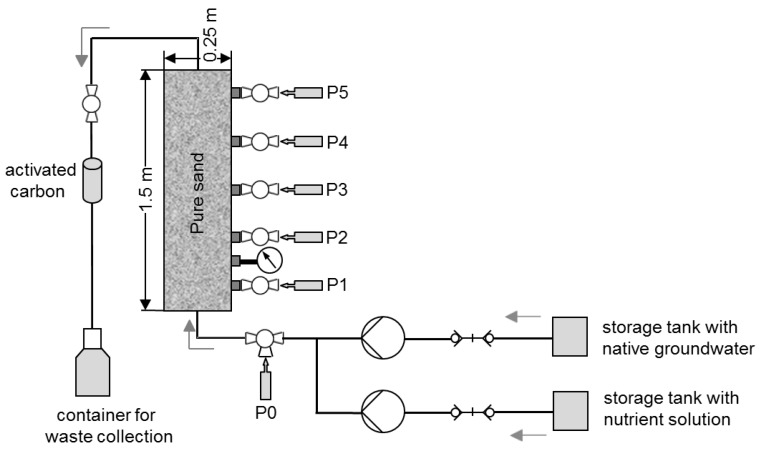
Experimental setup.

A one-dimensional vertical stainless steel column (length of 1.5 m and inner diameter of 0.25 m) was packed with uncontaminated soil (pure sand, K = 3 × 10^−4^ m/s) from the site and subjected to a flow of contaminated water for approximately 66 months (Figure 1). Water samples were collected from five slots distributed at equal distances over the whole column length (sampling points P1 to P5) and from one slot installed in the inflow of the column (sampling point P0). The column was operated in up-flow mode to prevent channeling and differential gravity flow. The hydraulic effective porosity was determined with 32 percent by a tracer test corresponding to a pore volume of 21 liters.

The column experiment was done in two steps: 

(1) Five liters of inoculum solution containing indigenous microorganisms from the most active batch experiments, electron acceptor nitrate, macro-nutrient phosphate, and trace elements were infiltrated into the column over a period of 24 h. It was followed by a 17-day period for the establishment of the microbial consortium. 

(2) After inoculation, native groundwater collected from the study area was infiltrated. The process parameters temperature, residence time of the infiltrated water (different residence times were realized by intermittent operation), as well as type and concentration of the used electron acceptors were varied during the different phases of the experiment. The process parameters were varied to observe their influence on the TMB degradation and to determine the optimal conditions for TMB degradation (Table 4). 

At first, a temperature of 20 °C was realized to show the potential of degradation of TMB isomers in principle (experimental phase 1, 2, 4, and 5). In the further course of the experiment, the column was cooled down (10–12 °C) to prove the degradation under groundwater conditions found at the contaminated site (experimental phase 3). The residence time in experimental phase 1 was chosen according to the flow rate at the site. It was increased from experimental phase 2 to give more time for degradation processes. The amount of infiltrated nitrate was increased from 120 mg/L to 200 mg/L and later to 300 mg/L during experimental phase 1, because of the increased consumption of nitrate due to higher biological activity.

### 2.5. Analytics

The aromatic hydrocarbon analyses were performed by using the headspace method according to the International Organization for Standardization ISO 11423-1 [23]. A 6890 gas chromatograph (Hewlett-Packard Company, Wilmington, DE, USA) equipped with a split/splitless injection port, a 0.53 mm × 29.8 m DB624 capillary column with a film thickness of 3 μm, and a flame ionization detector was used. Ten milliliters of each water sample was transferred to a 20-ml headspace vial using an Eppendorf pipette. The vials were closed immediately with gastight caps and thermostatted at 80 °C for 3 h. Afterwards, 250 µl of the gas volume was injected automatically (split ratio: 1:2.5) into the GC and analyzed. The chromatographic conditions were as follows: injection port temperature 250 °C, initial column temperature 90 °C, initial time 10.5 min, heating rate 5 °C/min, final temperature 250 °C, final time 1.5 min, column flow rate 4 mL/min helium. The detection limits for all identified compounds varied from 0.005 mg/L to 1 mg/L. 

The ions nitrate, nitrite, sulfate, and phosphate were analyzed according to International Organization for Standardization ISO 10304-1 [24]. A separation Center 733 (Metrohm Company, Filderstadt, Germany) equipped with the Metrosep separation column Chrompack 7414 (4.6 × 75 mm) and a conductivity detector were used for the analyses. Detection limits were 0.5–25 mg/L for nitrate, sulfate, and phosphate and 0.1–25 mg/L for nitrite.

### 2.6. Calculation of Degradation Rates

The calculation of degradation rates was performed using the first-order decay rate law [25].

$$A=A_{0}\times e^{-k\times t}$$

where

A_0_ = initial molar concentration of reactant A (mol/L)
A = molar concentration of reactant (mol/L)
k = rate constant (s^−1^)
t = time (s)

It was assumed that the reduction of pollutants took place under constant boundary conditions after a certain period of adaptation. The first-order decay rate is a simplification to replicate microbial kinetics, because an even distribution of bacteria was adopted. 

### 2.7. Calculation Mass Balance

The plausibility of the nitrate and sulfate consumption for the degradation of infiltrated organic substances was analyzed by a balance between inflow and outflow of the column. To get a complete mass balance, it was carried out for BTEX and TMB together. Theoretical required amounts of nitrate or sulfate for the mineralization of 1 mg of the individual substances were calculated according to Table 5. 

The column “Complete denitrification” represents here the required amount of nitrate in the case of the complete reduction of nitrate to N_2_, whereas “Incomplete denitrification” shows the required amount of nitrate if the reduction of nitrate stops after the formation of nitrite. Complete denitrification of BTEX and TMB to nitrogen follows the stoichiometry of reaction equations displayed exemplary for TMB:

$$C_{9}H_{12}+9.6NO_{3}^{-}+18H_{2}O+9.6H^{+}\to 9CO_{2}+28.8H_{2}O+4.8N_{2}$$

In the case of incomplete denitrification to nitrite, the following reaction equation describes the stoichiometry exemplary for TMB:

$$C_{9}H_{12}+24NO_{3}^{-}+18H_{2}O\to 9CO_{2}+24H_{2}O+24NO_{2}^{-}$$

In the case of TMB degradation under sulphate-reducing conditions, the following reaction equation describes the stoichiometry:

$$C_{9}H_{12}+6SO_{4}^{2-}+45H^{+}\to 9CO_{2}+6H_{2}S+4.8H_{2}O$$

## 3. Results and Discussion

### 3.1. Microcosm Experiments

The average measured concentrations in active batches were corrected by the average concentration of abiotic controls. The concentrations of aromatic compounds in abiotic controls were relatively stable; maximum reduction compared to the initial concentration was below 15%. Results are represented as normalized values to the initial concentrations. 

#### 3.1.1. Electron Acceptor Nitrate

**Figure 2:**
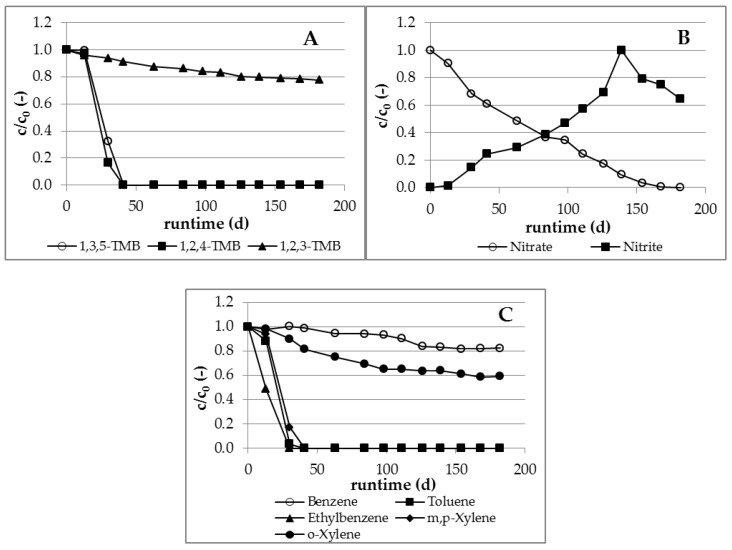
Degradation of TMB and BTEX under denitrifying conditions: (**A**) TMB, (**B**) Nitrate + nitrite, (**C**) BTEX.

Complete elimination of 1,2,4-TMB and 1,3,5-TMB within 40 days was obtained in all of the five active microcosms under denitrifying conditions (Figure 2A). 1,2,3-TMB was found to be less degradable, with only 22% degraded in the runtime. The degradation of TMB occurred in parallel with the degradation of BTEX contained in the water (Figure 2C). The compounds toluene, ethylbenzene, and m,p-xylene were completely degraded within 40 days. Only a partial degradation was observed for benzene (18%) and o-xylene (41%).

The reduction of pollutants correlated very well with the consumption of nitrate and the formation of nitrite up to a concentration of 179 mg/L (Figure 2B). Nitrite was accumulated temporarily as a result of incomplete denitrification. Nitrite was reduced to nitrogen by the occurring degradation processes after the complete consumption of nitrate (about 150 days). High nitrite concentrations could be one reason for the inhibition of 1,2,3-TMB, benzene and o-xylol biodegradation from 100 days of experimental runtime. These results are consistent with results reported by Burland and Edwards [26], where reduction of nitrate only to nitrite inhibited the degradation of BTEX (e.g., benzene).

#### 3.1.2. Electron Acceptor Sulfate

**Figure 3:**
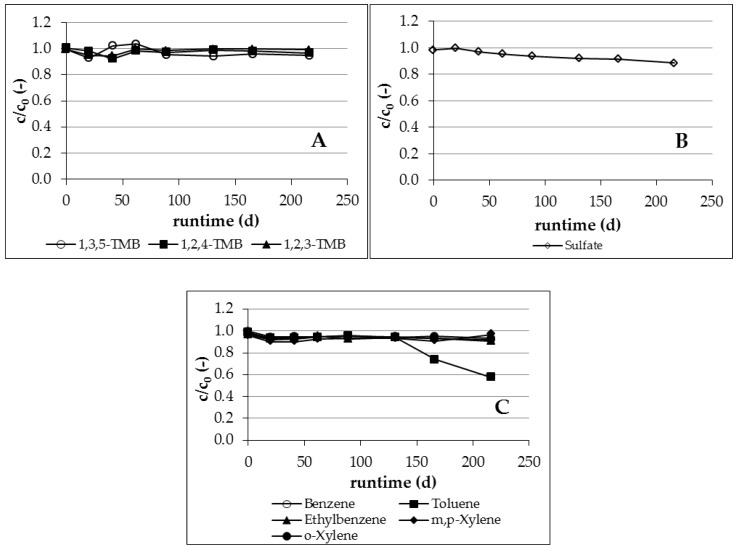
Degradation of TMB under sulfate-reducing conditions: (**A**) TMB, (**B**) Sulfate, (**C**) BTEX.

No degradation of all TMB isomers was detected in the five active microcosms under sulfate-reducing conditions over the runtime of 216 days (Figure 3A). This assumption is supported by the fact that only toluene, as representative of BTEX, was degraded under these conditions (Figure 3C).

Particularly, the lack of degradation of 1,3,5-TMB and 1,2,4-TMB is unexpected, given the positive results from other laboratory studies [11,12,14]. The beginning of toluene degradation at the end of the experimental runtime indicates that the lag-phase for degradation of other BTEX and TMB isomers possibly was too short. The undetectable degradation may also be caused by the relatively low temperature, since a successful degradation reported by Thierrin et al. [15] took place at 21 °C. The non-existing biodegradation of pollutants correlated with almost no consumption of sulfate in all five active approaches (Figure 3B).

### 3.2. Column Experiment

#### 3.2.1. Biodegradation of TMB-Isomers

##### 1,3,5-TMB—Electron Acceptor Nitrate (Experimental Phase 1 to 4)

**Figure 4:**
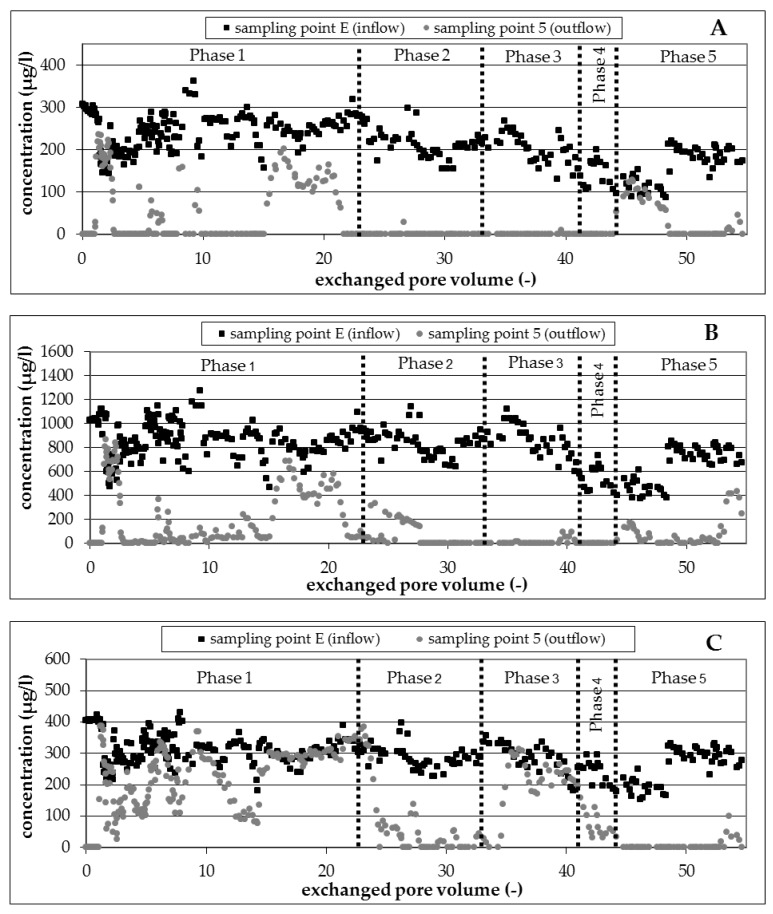
Degradation of 1,3,5-TMB (**A**), 1,2,4-TMB (**B**), and 1,2,3-TMB (**C**) under denitrifying and sulfate-reducing conditions.

**Figure 5:**
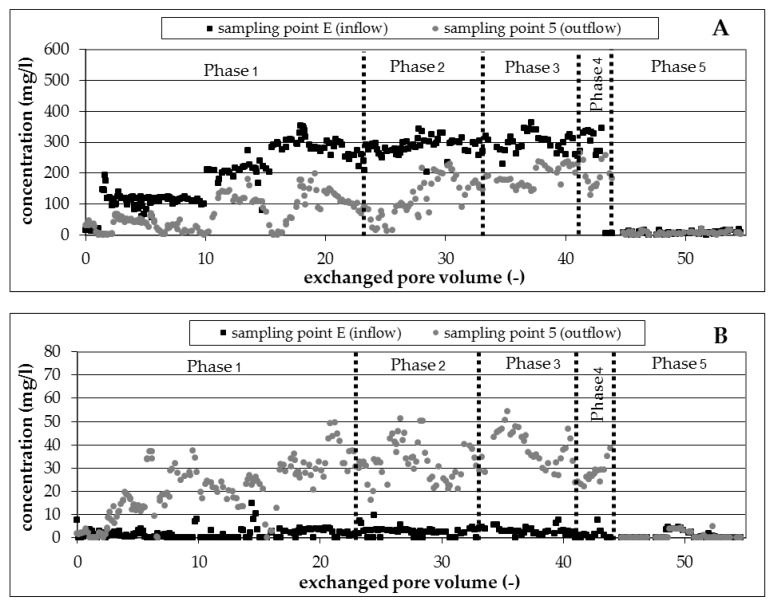
Concentration profile of nitrate (**A**) and nitrite (**B**) in the runtime of the column experiment.

The isomer 1,3,5-TMB proved to be well degradable under denitrifying conditions independent from residence time and temperature in the experimental phases 1 to 4 (Figure 4A); a nearly complete degradation was observed at sampling point 5. An exception was observed only between pore volumes 15 to 21.6 (experimental phase 1), due to a reduced availability of nitrate (Figure 5A). The infiltrated nitrate (200 mg/L) was consumed completely at exchanged pore volume 15 due to high biological activity and associated complete degradation of all BTEX/TMB. The degradation came partly to a standstill due to the insufficient amount of available nitrate. The degradation started again and was complete after the increase in the amount of infiltrated nitrate to 300 mg/L (Table 4) and a lag-phase of 6 exchanged pore volumes. 

##### 1,3,5-TMB—Electron Acceptor Sulfate (Experimental Phase 5)

Contrary to the observations in microcosms experiments, where a degradation of 1,3,5-TMB could not be demonstrated, it was well degradable under sulfate-reducing conditions (Figure 4A). A complete degradation was registered after a lag-phase of 5 exchanged pore volumes because of changed redox conditions at the beginning of experimental phase 5. 

Results observed by the experiments, as well as reported by Thierrin et al. [15], indicate that the biodegradation of 1,3,5-TMB is highly temperature-dependent. In the case of lower temperatures (10 °C), as with the microcosm experiments, microorganisms were observed to need a longer lag phase before starting degradation of 1,3,5-TMB.

##### 1,2,4-TMB—Electron Acceptor Nitrate (Experimental Phase 1 to 4)

Nearly complete degradation of 1,2,4-TMB was observed at sampling point 5 independent of the boundary conditions residence time and temperature in the experimental phases 1 to 4 (Figure 4B). A decline of degradation due to an insufficient amount of available nitrate was observed from exchanged pore volume 15 to 21.6. Only 30 percent of infiltrated 1,2,4-TMB was degraded in this phase. The observations of the present investigation confirm previously reported results [2,3,7,8,9,10,27]. 

##### 1,2,4-TMB—Electron Acceptor Sulfate (Experimental Phase 5)

1,2,4-TMB was well degradable under sulfate-reducing conditions (Figure 4B), which is consistent with the observations for 1,3,5-TMB. The higher temperature in comparison to the microcosm experiments also seems to provide more suitable conditions for microbial activity corresponding with better biodegradation. No lag-phase was registered after changing the electron acceptor from nitrate to sulfate, as was also observed for 1,3,5-TMB. A decline of degradation was observed at the end of experimental phase 5 (from exchanged pore volume 53). Only 50 percent of infiltrated 1,2,4-TMB was degraded in this phase. This behavior could not be explained, as boundary conditions were not changed and sufficient sulfate was available.

##### 1,2,3-TMB—Electron Acceptor Nitrate (Experimental Phase 1 to 4)

Contrary to the results for well degradable compounds 1,3,5-TMB and 1,2,4-TMB, the degradation of 1,2,3-TMB was not constant. However, partial degradation was observed during the infiltration of native ground water for several time intervals (Figure 4C). Depending on the applied conditions (Table 4), phases with a continuous, complete degradation alternated with phases of incomplete degradation. A partial degradation of 1,2,3-TMB up to 70 percent was registered in experimental phase 1 at a temperature of 20 °C and a residence time of 16 days, but the degradation was not continuous in this phase. The temporary incomplete degradation was stopped by the lack of electron acceptor nitrate after 15 exchanged pore volumes (Figure 5A). A degradation of 1,2,3-TMB was registered again in experimental phase 2, corresponding with the increase in residence time from 16 to 29 days. With few exceptions, it can be seen that the degradation was nearly complete in this phase. More time was available to destabilize the aromatic ring with the vicinal structure because of the higher residence time of 1,2,3-TMB on the way through the column. However, the degradation came to a standstill with the lowering of the temperature from 20 °C to 10 °C. The biological activity here was not sufficient to destabilize the aromatic ring and to degrade the 1,2,3-TMB. Degradation was detected again in phase 4 with the increase of temperature (Table 4) and the associated higher biological activity. 

##### 1,2,3-TMB—Electron Acceptor Sulfate (Experimental Phase 5)

1,2,3-TMB was well degradable under sulfate-reducing conditions (Figure 4C). No lag-phase was registered after changing the electron acceptor from nitrate to sulfate. This result was not to be expected, due to the non-existent evidence of biodegradation under sulfate-reducing conditions in the literature and in the performed microcosm experiments. The addition of nitrate followed by sulfate was probably the trigger for the identified biodegradation of 1,2,3-TMB under sulfate-reducing conditions. This stimulation effect could also be observed during the biodegradation of other hydrocarbons [28].

#### 3.2.2. Biodegradation of BTEX

##### BTEX—Electron Acceptor Nitrate (Experimental Phase 1 to 4)

The degradation of BTEX occurred in parallel with the degradation of TMB contained in the water. The compounds toluene, ethylbenzene, and m,p-xylene were very degradable independent of the boundary conditions and were completely reduced. The degradation behavior was comparable to those of 1,3,5-TMB and 1,2,4-TMB. Partly, incomplete degradation was observed for benzene and o-xylene—the degradation behavior was the same as for 1,2,3-TMB. The results are consistent with those observed in the microcosm experiments.

##### BTEX—Electron Acceptor Sulfate (Experimental Phase 5)

All the BTEX compounds, except benzene (from exchanged pore volume 53), were well degradable under sulfate-reducing conditions. A complete degradation was registered as observed for all the TMBs in the column experiments. The results are contrary to those observed in the microcosm experiments, in which no degradation of any TMB and BTEX compounds, except toluene, was detected under sulfate-reducing conditions. The reason for this observation could be again the higher temperature in the column experiment, conducive for high microbial activity, and the destabilization of the aromatic ring.

#### 3.2.3. Degradation Rates

Degradation rates were calculated for the entire column length (sampling point P0 to sampling point P5) for the five different experimental phases (Table 6). In addition, the rates were determined for the five successive column sections (sampling point P0 to sampling point P1, sampling point P1 to sampling point P2, etc.) to identify zones with the highest degradation rates and biological activity. The bases for the calculation were the mean concentrations in the inflow and outflow of the column or column sections for the five experimental phases.

It was demonstrated that the highest degradation rates and thus the highest biological activities under denitrifying conditions in phases 1 to 4 can be found between sampling point E and 2. The calculated degradation rates for 1,2,4-TMB in the phases with denitrifying conditions are in the same range as the rate (0.189 [1/d]) determined by experiments from Kao et al. [27] as well as the rate (0.148 to 0.27 [1/d]) reported by experiments from Hutchins et al. [2]. The lowering of the temperature in experimental phase 3 had no influence on the degradation rate of 1,2,4-TMB and 1,3,5-TMB. In comparison, there was a strong decline in the rate of degradation for 1,2,3-TMB because of the lowering of the temperature and the associated lower biological activity in experimental phase 3. Degradation rates for 1,3,5-TMB and 1,2,4-TMB for the entire column length under sulfate-reducing conditions were significantly lower than those under denitrifying conditions. In contrast to this result, the degradation rate of 1,2,3-TMB, poorly degradable under denitrifying conditions, increased under sulfate-reducing conditions. Further, it is noticeable that relatively high degradation rates were determined in the upper column horizons for the experimental phase 5 (P3-P4, P4-P5) for 1,2,4-TMB and 1,2,3-TMB.

#### 3.2.4. Ions

##### Nitrate and Nitrite

The reduction of pollutants correlated with the consumption of nitrate and the formation of nitrite during experimental phases 1 to 4 (Figure 5A,B). An unbalanced nitrate consumption from 30 mg/L (experimental phase 1) to 200 mg/L (experimental phase 1 and 2) was observed. 

Temporary accumulation of nitrite up to 50 mg/L in phases with an excess of nitrate provided clear evidence for biological activity and denitrifying processes in the column. The inhibition of biodegradation by high nitrite concentrations could not be observed. Nitrite was reduced to nitrogen by the occurring degradation processes if the infiltration of nitrate was stopped (Figure 5B, experimental phase 5). 

##### Sulfate

**Figure 6:**
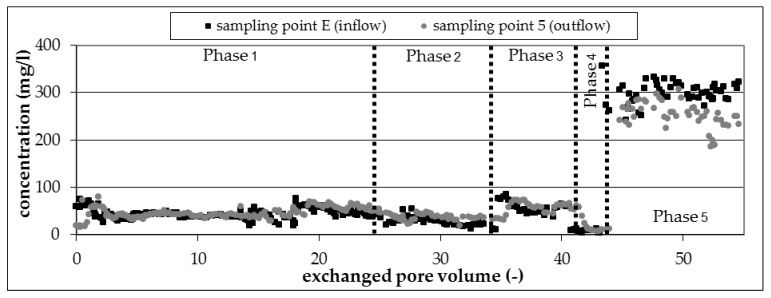
Concentration profile of sulfate in the runtime of the column experiment.

The consumption of sulfate was observed after changing the electron acceptor from nitrate to sulfate in experimental phase 5 (Figure 6). However, the consumption was low at the beginning because of the missing or incomplete degradation of some BTEX and TMB compounds. Sulfate consumption increased later up to 50 mg/L with increasing degradation of pollutants during experimental phase 5.

#### 3.2.5. Mass Balance

Mass balance for experimental phases 1–5 was calculated for BTEX and TMB together (Table 7). It was assumed that approximately 60 percent of the BTEX and TMB compounds were completely mineralized whereas the rest was used for biomass production. Incomplete denitrification to the intermediate level nitrite in experimental phase 1 to 4 was one reason for the increased consumption of nitrate compared to the theoretical demand. Moreover, the remaining nitrate (47.2 g) in experimental phases 1–4 and sulfate (2.7 g) in experimental phase 5 was used for the oxidation of other organic contaminants (PAHs, alkylphenols) contained in the water. Further consumption of nitrate is caused by anaerobic oxidation of inorganic compounds, such as pyrite (FeS_2_) and Fe[II]-silicates utilized by denitrifying organisms [29]. The pyrite oxidation leads to sulfate production which explains the sulfate production in experimental phases 1 to 4. 

## 4. Conclusions

The results obtained from the experiments demonstrate that indigenous microorganisms are in principle able to degrade TMB isomers under denitrifying and sulfate-reducing conditions at the study site. The length of lag phases and the degree of biodegradation are mainly dependent on the boundary conditions temperature, residence time, and availability of electron acceptors. 

The investigations also indicate that biodegradation of 1,2,4-TMB and 1,3,5-TMB under denitrifying conditions in principle is preferred, which was demonstrated by a continuous, complete degradation after shorter lag phases. Changes in temperature and residence time have little to no influence on the biodegradation of these TMB isomers. Contrary to that, the biodegradation of 1,2,3-TMB after longer lag-phases was not continuous—phases with a complete degradation alternated with phases with incomplete degradation. The biodegradation of 1,2,3-TMB was more sensitive to changes in temperature and residence time, whereby higher temperatures were conducive to biodegradation processes. The reason for the different levels of biodegradability under anaerobic conditions is the structure of the TMB isomers. More time combined with higher biological activity is necessary for destabilization of the aromatic ring of 1,2,3-TMB because of the vicinal structure.

The biodegradability of all TMB isomers under sulfate-reducing conditions was strongly dependent on temperature. Continuous and complete degradation was observed at higher temperatures (20 °C) for all TMB isomers, whereby no degradation was observed at lower temperatures (10 °C). 

In conclusion, the results suggest that the use of TMB as a conservative tracer for the evidence of BTEX attenuation should only be applied with caution under denitrifying and sulfate-reducing conditions. The biodegradation of TMB under anaerobic conditions is site specific and therefore preliminary studies are necessary before the use of TMB isomers as conservative tracers. The outcomes of this study can be used to prevent the underestimation of BTEX biodegradation at field sites.

## Figures and Tables

**Table 1:** Composition of the used contaminated groundwater (average concentrations and the minimum/maximum concentrations of pollutant loads/electron acceptors in parentheses). Listed concentrations are in mg/L.

Benzene	Toluene	Ethyl-benzene	m,p-Xylene	o-Xylene	1,3,5-TMB	1,2,4-TMB	1,2,3-TMB	NO_3_^−^	SO_4_^2−^
1.5 (0.6/2.5)	4.0 (2.2/6.9)	1.5 (1.2/2.5)	3.8 (1.4/6.4)	1.3 (0.7/2.8)	0.2 (0.08/0.36)	0.8 (0.4/1.3)	0.3 (0.2/0.5)	8 * (4/16)	40 * (33/45)

* Native concentration; for the additional amounts of nitrate and sulfate, see Table 3. TMB: trimethylbenzene.

**Table 2:** Composition of the trace element solution (TES), concentrations in mg/L.

ZnSO_4_·7H_2_O	100	CaCl_2_·H_2_O	197	FeSO_4_·7H_2_O	100	AlK(SO_4_)_2_·12H_2_O	10
NiCl_2_·6H_2_O	10	H_3_BO_3_	20	MgSO_4_·7H_2_O	3.0		
CuSO_4_·5H_2_O	10	Na_2_MoO_4_·2H_2_O	100	Na_2_WO_4_·2H_2_O	10		
MnSO_4_·H_2_O	500	Co(NO_3_)_2_·6H_2_O	10	Na_2_SeO_3_	1		

**Table 3:** Composition of the microcosm experiment, concentrations in mg/L.

	Total BTEX	1,3,5-TMB	1,2,4-TMB	1,2,3-TMB	NO_3_^−^	SO_4_^2−^	PO_4_^3−^	TES	NaN_3_
Nitrate microcosm	14.1	0.26	0.91	0.33	200	40 *	30	3	1500 **
Sulfate microcosm	14.1	0.26	0.91	0.33	8	300	30	3	1500 **

* Native concentration; ** Poisoned control only. BTEX: benzene, toluene, ethylbenzene, and xylenes.

**Table 4:** Characterization of boundary conditions in the column experiment.

Temp. (°C)	Exchanged Pore Volumes (EPV) (-)	Residence Time (d)	Flow Velocity (m/s) or (m/a)	Addition of Electron Acceptors/Macro Nutrients after x EPV	Exp. Phase
20	0.0–23.2	16	1.09 × 10^−6^ or 34.2	from EPV 0 120 mg/L nitrate 30 mg/L phosphate 3 mg/L TES from EPV 8.4 200 mg/L nitrate 60 mg/L phosphate 3 mg/L TES from EPV 14.1 300 mg/L nitrate 60 mg/L phosphate 3 mg/L TES	1
	23.2–32.7	29	6.0 × 10^−7^ or 18.8	No changes	2
10–12	32.7–40.6	29	6.0 × 10^−7^ or 18.8	No changes	3
20	40.6–43.0	29	6.0 × 10^−7^ or 18.8	No Changes	4
	43.0–54.8	29	6.0 × 10^−7^ or 18.8	from EPV 43.0 300 mg/L sulfate 60 mg/L phosphate 3 mg/L TES	5

**Table 5:** Required amounts of nitrate and sulfate for mineralization, concentrations in mg/mg.

	Complete Denitrification	Incomplete Denitrification	Sulfate-reducing Conditions

Benzene	4.77	11.9	4.61
Toluene	4.85	12.1	4.7
Ethylbenzene	4.91	12.3	4.75
Xylene	4.91	12.3	4.75
TMB	4.96	12.4	4.8

**Table 6:** Degradation rates k (1/d) of TMB isomers calculated based on the results of column experiments.

		P0-P5	P0-P1	P1-P2	P2-P3	P3-P4	P4-P5
**Phase 1**	1,3,5-TMB	0.09	0.49	0.05	0.08	0.02	0.03
	1,2,4-TMB	0.09	0.42	0.05	0.06	0.04	0.03
	1,2,3-TMB	0.02	0.04	0.04	0.004	<0.01	0.04
**Phase 2**	1,3,5-TMB	0.20	0.61	0.37	<0.01	0.14	0.14
	1,2,4-TMB	0.08	0.49	0.14	0.01	<0.01	0.02
	1,2,3-TMB	0.04	0.15	0.11	0.02	<0.01	<0.01
**Phase 3**	1,3,5-TMB	0.21	1.33	n.d.	n.d.	n.d.	n.d.
	1,2,4-TMB	0.19	1.03	0.28	0.04	<0.01	0.28
	1,2,3-TMB	0.03	0.05	0.05	0.04	0.04	0.04
**Phase 4**	1,3,5-TMB	0.18	0.93	0.15	n.d.	n.d.	n.d.
	1,2,4-TMB	0.16	0.81	0.13	<0.01	<0.01	0.17
	1,2,3-TMB	0.01	0.10	0.003	<0.01	<0.01	0.01
**Phase 5**	1,3,5-TMB	0.05	0.22	0.03	0.04	0.04	0.01
	1,2,4-TMB	0.07	0.02	0.02	0.01	0.11	0.15
	1,2,3-TMB	0.11	0.04	0.03	0.02	0.19	0.21

Note: n.d.—not detectable.

**Table 7:** Mass balance for experimental phases 1–5, mass in g.

	Phase 1–4	Phase 5
BTEX/TMB inflow	11.8	2.7
BTEX/TMB outflow	2.0	0.3
BTEX/TMB mineralized	5.9	1.5
Nitrate inflow	218.8	1.7
Nitrate outflow	100.3	1.4
Nitrate consumed	118.5	0.3
Theoretical demand of nitrate for complete denitrification	28.5	7.2
Theoretical demand of nitrate incomplete denitrification	71.3	18.1
Sulfate inflow	37.2	70.8
Sulfate outflow	38.1	61.2
Sulfate consumed	−0.9	9.6
Theoretical demand of sulfate	27.1	6.9
